# Performance and modification of EGCRISC among hepatitis C virus high-risk groups

**DOI:** 10.1017/S0950268820000175

**Published:** 2020-02-14

**Authors:** E. M. El-Ghitany, Y. M. Alkassabany, A. G. Farghaly

**Affiliations:** Tropical Health Department, High Institute of Public Health, Alexandria University, Alexandria, Egypt

**Keywords:** EGCRISC, HCV, HIV, IDUs, risk groups

## Abstract

We have previously shown that the Egyptian Hepatitis C Virus Risk Score (EGCRISC), an Egyptian hepatitis C virus (HCV) risk-based screening tool, to be valid and cost-effective. Certain behaviours, occupations and diseases have been shown to be associated with an increased risk of exposure to HCV infection and constitute a major population reservoir of HCV infection. This study investigated the efficacy of EGCRISC in selected high-risk groups by testing 863 participants from four groups: slaughterhouse workers, illicit drug users (IDUs), female sex workers and human immune deficiency virus (HIV) patients. Data for this study were collected on EGCRISC and another pre-designed risk factor questionnaire. Sera were tested for HCV antibodies by ELISA. EGCRISC, at lower cut-off points, showed significantly good performance (*P* < 0.05) in all four groups except for females <45 years, but was reliable in detecting HCV cases (sensitivity: 84.21% and negative predictive value: 94.5%). Specific scores for IDUs and HIV patients were developed that showed high accuracy (*P* < 0.001). A modified EGCRISC for high-risk groups (EGCRISC-HRGs) was shown to be a valid tool that is recommended for use in high-risk populations if no other specific screening tool is available or universal screening is applied. EGCRISC for IDUs (EGCRISC-IDUs) and EGCRISC for HIV patients (EGCRISC-HIV) are useful tools for preselecting potentially HCV-infected cases for further testing in settings where serological analysis is not readily available or accessible.

## Introduction

The World Health Organization (WHO) has recently formulated the ‘Global Health Sector Strategy on Viral Hepatitis’. Elimination of hepatitis C virus (HCV) as a public health threat by 2030 is one of its targets [1].

Egypt is one of the countries that have the highest HCV burden [2]. The prevalence of HCV antibody was estimated at 14.7% in 2008 [3] and at 10% in 2015 [4]. Extensive efforts to control HCV have been initiated at the national level. This has generated strong international support [5]. The Egyptian National Committee for the Control of Viral Hepatitis has developed a country-wide strategy for the control of viral hepatitis. Four priority areas were identified: surveillance and monitoring, prevention, patient management and research. This was followed by the Plan of Action for the Prevention, Care and Treatment of Viral Hepatitis developed in 2014–2018 [6].

Despite all Egyptian efforts supported by WHO and other agencies, the main focus until recently has been a national programme for the treatment of HCV patients [7]. It is essential for the eradication of HCV in Egypt to adopt other prevention and control measures. Due to the long asymptomatic phase of HCV infection, early detection is the challenge in HCV control. This can only be achieved through screening [8].

Accordingly, we have developed a valid, specific and cost-effective EGCRISC screening tool that can be used as a first-level approach to identify persons who have a high probability of being HCV infected and consequently should undergo further serologic testing. EGCRISC scores categorise the general population into low risk (green zone), moderate risk (yellow zone) or high risk (red zone) [8–11].

However, certain groups of behaviours, occupations and diseases likely to be associated with an increased risk of exposure to HCV infection may require a modified EGCRISC score as these groups were not included at the time EGCRISC was first developed and validated. Individuals who may be potentially at high risk need to be selectively targeted. Moreover, special risk groups in Egypt such as illicit drug users (IDUs), human immune deficiency virus (HIV) patients, men who have sex with men (MSM) and female sex workers (FSWs) are generally hidden, hard to reach, underestimated and underscreened due to social stigma and the holistic approach of the Egyptian research organisations for the screening of HCV. In addition, HCV screening has not been included among people at greater risk owing to their occupation such as health care workers and slaughterhouse workers (SHWs). SHWs have been shown to be at risk of acquiring and transmitting blood-borne infections from cuts and blood-letting [12].

In this study, we tested the efficacy of EGCRISC against HCV antibody positivity in subgroups in the Egyptian communities, specifically IDUs, HIV patients, SHWs and FSWs that have often been excluded but may be at an increased likelihood of HCV exposure and infection. This study aimed at testing the performance of EGCRISC with and without modification as needed.

## Subjects and methods

### Study subjects and settings

Four high-risk groups namely FSWs, HIV patients, IDUs and SHWs were included in the study. This was a cross-sectional study, and participants were recruited from the following different settings

- Five randomly chosen slaughterhouses distributed in four governorates: Cairo, Alexandria, Beheira and Gharbia.
- Drug users treatment and rehabilitation centres:
  - Freedom centre for treatment of addiction and AIDS, IKingi Mariut branches in Alexandria.
  - Addiction treatment centre at Al-Abbassya hospital in Cairo.
  - Addiction treatment centre at El-Maamoura hospital in Alexandria.
- HIV clinic in Alexandria fever hospital.
- Al-Shehab institution for comprehensive development in Cairo which provides service and care for women with high-risk behaviours including addiction and commercial sex work.

The criteria for inclusion were as follows:

- at least 16 years old.
- evidence of risky behaviours during the preceding year of enrolment.
  - For SHWs: working at a slaughterhouse.
  - For IDUs: injecting and/or non-injecting drug user.
  - For HIV patients: confirmed both serology (HIV1 or HIV2 antibody) and PCR or on antiretroviral treatment.
  - For FSWs: females with a history of commercial sex work.

Individuals from each of these groups were selected by simple random sampling except for HIV patients who were recruited consecutively when fulfilling the inclusion criteria.

### Data collection method

A face-to-face interview was conducted to fill the EGCRISC form with the predetermined cut-off points and zone limits: green, yellow and red zones for low-, intermediate- and high-risk individuals, respectively. Other potential risk factors were also inquired about [8, 11].

The questionnaire included information on socio-demographic data, community-acquired risk factors, medical and iatrogenic risk factors, behavioural risk factors, risk factors related to the partner, non-specific unexplained fatigue during the previous 6 months and risk behaviours specific to each group including duration and age at start of risk practice, sex behaviours, injection practices in IDUs and occupational practices in SHWs.

Two samples of 5 ml venous blood were obtained from all study subjects and collected into two labelled clean dry tubes without an anticoagulant. Samples were allowed to clot and sera were separated by centrifugation at room temperature then were stored at –20 °C for analysis. Blood samples were tested within 24 h of the collection at Mabaret ElAsafra Laboratory for HCV antibody by commercial Enzyme Linked Immunosorbent Assay (3rd generation ELISA kits; DIALAB^®^). A second serum sample was retested by ELISA (Murex^®^) for confirmation and possible false-positive results.

### Ethical considerations

The study was done according to the standard international ethical guidelines. Approval was taken from the ethics committee of the High Institute of Public Health (HIPH). Official approval of relevant authority was taken before the start of the study. Participants were recruited after getting written informed consent.

### Statistical analysis

Statistical analysis was done using IBM Statistical Package for the Social Sciences (SPSS) statistics program version 21 and Medcalc programs. Quantitative data were described by mean and as a measure of central tendency and by standard deviation, minimum and maximum as measures of dispersion, while categorical variables were summarised by frequency and per cent. The *χ*^2^ test was used to test for a significant association between two categorical variables. Fisher's exact and Montecarlo tests of significance were used if more than 20% of the total expected cell counts <5.

For determining variables to be included in the newly developed tools for HIV patients and IDUs, multivariate stepwise logistic regression using backward (Wald and Likelihood ratio) methods was used and included only the statistically significant risk factors based on bivariate analysis. The Wald and Likelihood ratio maximises the log likelihood on how likely the observed grouping can be predicted from observed values of predictors.

Hosmer and Lemeshow test was used to assess whether the predicted probabilities match the observed probabilities using a *P*-value < 0.05. Nagelkerke *R*^2^ was calculated to explain the amount of variance in HCV outcome accounted by the model. Standardised residuals and Cook's distance were calculated to test for the presence of outliers with influential cases. Testing for the presence of multicolinearity by correlation of estimates and standard error of regression coefficients was carried out.

Variables were scored by their magnitude of association (odds ratio), and those without significant contribution were sequentially removed from the model until all variables reached statistical significance in the full multivariate model. For each patient, the HCV risk score was calculated as the simple arithmetic sum of the nearest integral value assigned to each risk factor based on the multivariate adjusted risk relationship [13].

Receiver operation coefficient (ROC) curve analysis was done to detect the diagnostic accuracy of different calculated scores for the screening of HCV infection. Area under the curve (AUC), sensitivity (SE), specificity (SP), positive predictive value (PPV) and negative predictive value (NPV) were used to evaluate each index, where SE = true positive/(true positive + false negative), SP = true negative/(true negative + false positive), PPV = true positive/(true positive + false positive) and NPV = true negative/(true negative + false negative).

For significant results, AUC of 0.90–1 = excellent, 0.80–0.90 = good, 0.70–0.80 = fair, 0.60–0.70 = poor, 0.50–0.60 = fail. Cut-off point for each score was determined by Youden index [14].

EGCRISC was given to the whole population as one group to avoid statistical issues that could arise from smaller sample sizes after stratifying into groups and further stratification by age and gender and to extrapolate the conclusion on other risk groups. The cut-off points in all strata were re-tested based on maximum possible accuracy parameters.

All statistical tests were considered significant at *P* ⩽ 0.05.

## Results

There were 863 participants included in this study. Characteristics and socio-demographic data of the participants are shown in Table 1. Age ranged from 15 to 72 years, the majority were males (79.1%) and most of them were from urban residence (69.4%). IDUs, SHWs, HIV patients and FSWs constituted 37.7%, 36.8%, 19.5% and 6% of the studied population, respectively.

**Table 1.** Distribution of participants according to their socio-demographic characteristics

Variables	No.	%
Age in years		
Range	15–72 years	
Mean ± s.d.	35.37 ± 11.44	
Gender		
Male	683	79.1
Female	180	20.9
Residence		
Urban	599	69.4
Rural	154	17.8
Slums	110	12.7
Education		
Illiterate	179	20.7
Read and write	67	7.8
Primary	112	13.0
Preparatory	113	13.1
Secondary	217	25.1
University	174	20.2
Marital status		
Single	292	33.8
Married	476	55.2
Divorced	56	6.5
Widowed	39	4.5
Risk group		
SHWs	318	36.8
IDUs	325	37.7
HIV	168	19.5
FSWs	52	6.0
Total	863	100.0

### Validation of EGCRISC

The performance of the original EGCRISC by zone distribution is illustrated in Table 2. EGCRISC showed a very good performance in the groups as a whole, but was not statistically significant in females. However, 100% of anti-HCV-negative cases were in the green zone and one-third of positive HCV cases were in the red zone in females >45 years.

**Table 2.** Validation of EGCRISC zones in different strata

EGCRISC zones	Total	Anti-HCV ELISA				*P-*value^a^
		Negative		Positive		
		No.	%	No.	%	
Males <45 years (*n* = 541)						
Green zone	102	92	90.2	10	9.8	0.000
Yellow zone	245	199	81.2	46	18.8	
Red zone	194	116	59.8	78	40.2	
Total	541	407	–	134	–	
Males >45 years (*n* = 143)						
Green zone	66	59	89.4	7	10.6	0.000
Yellow zone	65	47	72.3	18	27.7	
Red zone	12	5	41.7	7	58.3	
Total	143	111	–	32	–	
Females <45 years (*n* = 152)						
Green zone	123	110	89.4	13	10.6	0.134
Yellow zone	25	19	76.0	6	24.0	
Red zone	4	4	100.0	0	0.0	
Total	152	133	–	19	–	
Females >45 years (*n* = 27)						
Green zone	26	24	92.3	2	7.7	0.120
Red zone	1	0	0.0	1	100.0	
Total	27	24	–	3	–	

a Test of significance: *χ*^2^ test.

EGCRISC for high-risk groups (EGCRISC-HRGs) was defined by lowering the original cut-off points for the four EGCRISC versions (by age and gender) according to the specific ROC for each one giving the best possible combination of sensitivity and specificity. EGCRISC-HRGs showed considerably good accuracy parameters at 100% sensitivity and 86.36% specificity in females >45 years. Accuracy parameters and the modified cut-off point for prediction of HCV antibody seropositivity are shown in Table 3.

**Table 3.** Accuracy parameters of EGCRISC among high-risk groups and of the new risk score of drug users and HIV patients

Stratum	AUC (s.e.)	*P*-value	Original cut-off	New cut-off point	Sensitivity	Specificity	PPV	NPV
Males <45 years	0.612	<0.0001	17	14	54.48	62.90	32.6	80.8
Males >45 years	0.687	0.0002	8	6	81.25	48.65	31.3	90
Females <45 years	0.537	0.5207	25	6	84.21	39.10	16.5	94.5
Females >45 years	0.947	<0.0001	15	8	100	86.36	50	100
EGCRISC-IDUs	0.911 (0.019)	0.001	18.5	–	88.54	83.15	73.9	93.1
EGCRISC-HIV	0.744 (0.0402)	<0.0001	9	–	30.77	96.55	80	75.7

HCV prevalence ranged from a low of 12% in females >45 years to a high of 24.8% in males <45 years. EGCRISC-HRGs was statistically significant for all groups except for females <45 years. In the latter group, the sensitivity and NPV were high (84.21% and 94.5%, respectively).

**Fig. 1.**
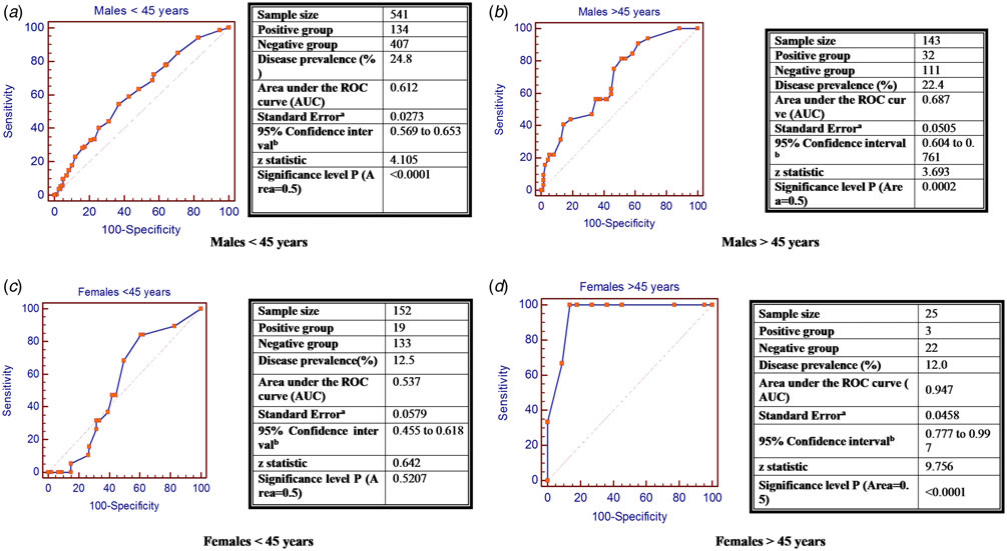
Receiver operating Characteristic curve for HCV seropositivity diagnostic accuracy of EGCRISC-HRGs and sensitivity and specificity at different cut-off values among the four gender/age different strata.

The ROC results in the four different strata categorised by EGCRISC are shown in Figure 1. ROC is statistically significant except for females <45 years.

### HCV risk assessment score for IDUs and HIV patients

**Fig. 2.**
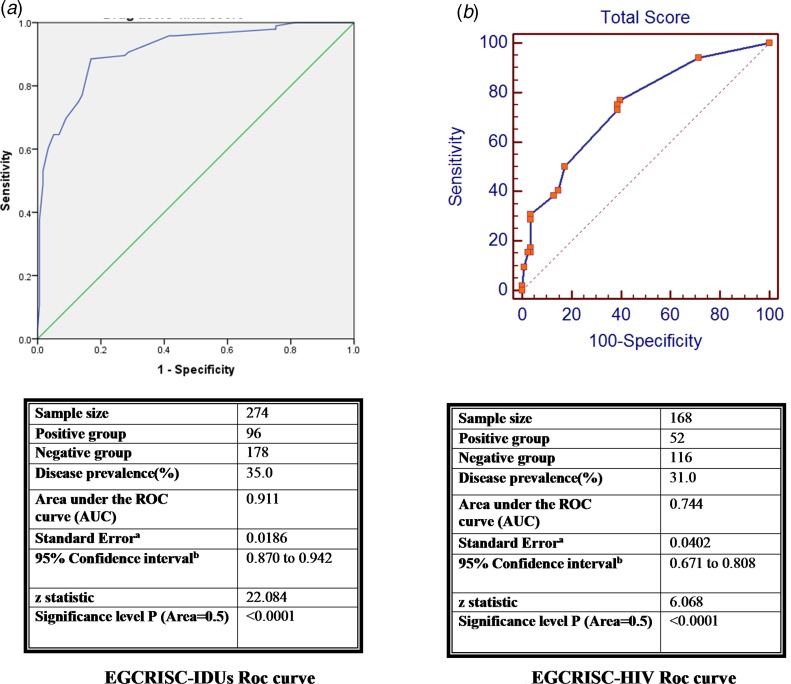
Receiver operating Characteristic curve for HCV seropositivity diagnostic accuracy of the new score among drug users (EGCRISC-IDUs) and HIV patients (EGCRISC-HIV).

The score and weights for each category of IDUs (EGCRISC-IDUs) and HIV patients (EGCRISC-HIV) are shown in Table 4. The ROC curves for both EGCRISC-IDUs and EGCRISC-HIV scores are illustrated in Figure 2. The cut-off points and the accuracy parameters are illustrated in Table 2. The EGCRISC-IDUs had an AUC (0.91) and a high accuracy as demonstrated by SE, SP, PPV and NPV at a cut-off point >18.5. The EGCRISC-HIV score also had significantly good accuracy parameters for predicting anti-HCV seropositivity in HIV patients. Specificity was 96.6% with considerable PPV and NPV although the sensitivity was relatively low.

**Table 4.** New risk scores for drug users and HIV patients

Risk score/items	Score
EGCRISC-IDUs	
History of imprisonment	3
Sharing needle/syringe	9
Injecting by others	3
Injecting for others	4
Using drug prepared in other syringes	3
Age 25–35 years	8
Age 35+ years	14
EGCRISC-HIV	
Gender (male)	3
Education (non-University)	3
Accidental puncture with protruding needle	4
History of imprisonment	5
Alcohol use	3

## Discussion

The EGCRISC-HRGs has the good discriminating ability for HCV-infected individuals particularly for males <45 years and females >45 years.

Several screening tools in different countries are currently available to screen for HCV on the basis of risk assessment [15–18]. McGinn *et al*. [15] found sensitivity and specificity of 34% and 97%, respectively, using a simplified tool based on the use of three domains for HCV risk factors.

The novel EGCRISC-HRGs was a modified score from the original EGCRISC by the reduction of cut-off points in all four strata. This is a logical and expected modification due to the baseline HCV risk in these groups. EGCRISC-HRGs has a discriminating ability for HCV antibody-seropositive individuals especially for males <45 years and females >45 years. Our results indicated that the highest sensitivity was among females >45 years which is consistent with the results of EGCRISC validation on the general Egyptian population [8]. EGCRISC-HRGs identified 100% of HCV-infected females aged above 45 years. EGCRISC-HRGs correctly identified 81.25% of HCV-infected males aged above 45 years. Despite not reaching a statistically significant level in females <45 years, EGCRISC-HRGs might be a useful tool in terms of detection of most HCV cases in this group due to high sensitivity and NPV.

Using EGCRISC for the screening of high-risk groups can potentially reduce unnecessary screening of people that are HCV-Ab negative and correctly identify those with low risk classified as green zone which included nearly one-quarter of seronegative males <45 to all seronegative females >45. Meanwhile, 7.5%, 21.9%, 66.7% and 68.4% could be missed from males <45, males >45, females >45 years and females <45 years, respectively.

The EGCRISC objective is to minimise missing subjects at the expense of accepting more false positives provided they are within acceptable limits for a risk-based screening tool. In this context, the NPV is the measure of choice for rule-out. NPV showed the best values in all the tools except in EGCRISC-HIV.

Comparing these results derived from EGCRISC validation on the general population [8], which showed the highest specificity (80%) among males >45 years, these results showed the highest (86.4%) among females >45 years. Additionally, the results were not significant for EGCRISC among females <45 years although still potentially useful particularly in terms of detection of most HCV cases [8]. Meanwhile, high false-positive rate (type I error) is expected. This may be attributed to the small sample size of this group included in the present study (152) in comparison to 1079 that were included in the previous report.

Nguyen *et al*. [17], using a self-administered 72-item questionnaire on 207 patients with unknown HCV status and 222 HCV-positive patients, reported a sensitivity and specificity of 24.4% and 99.4%, respectively, if four or more risk factors were present.

In the Netherlands [16], a study was developed in three phases: development of the content, formulation and testing of the core (18 items) and extended (20 items) risk assessment questionnaire on the public and finally its validation on patients attending a clinic for sexually transmitted infections (STIs). The extended questionnaire had a high sensitivity of 84.6% and increased to 90% by its application on STI clinic patients. The author concluded that their instrument succeeded in identifying at-risk individuals and recommended online application, especially in European countries where the prevalence of HCV is relatively higher than in the Netherlands.

The major advantages of EGCRISC include the small number of predictors ranging from 8 to 13 in each of the four stratified groups, two categorizing methods (cut-off and zones), high accuracy parameters relative to similar tools and applicability on general population as well as high-risk groups. Additionally, an electronic interactive version and a mobile application could be developed as has previously done for the original EGCRISC. This would facilitate self-screening in stigmatised groups together with health education campaigns. Paper versions with scores could be distributed at different facilities such as primary health care facilities, fever hospitals, institutes caring for any of the high-risk groups, national ID and driving license issuing offices.

The high prevalence of HCV among drug users, particularly people who inject drugs (PWIDs), in Egypt would justify universal HCV screening among them. Otherwise, application of the sensitive and specific EGCRISC-IDUs is recommended, and would improve the identification of HCV patients in this population.

A similar cross-sectional study was conducted in Australia [18] on 16 127 PWIDs in order to develop and validate a scoring tool that was based on socio-demographic as well as injecting risk behaviours to identify those who require additional serologic testing. The former tool demonstrated strong relationships between an individual's HCV score and their risk by antibody testing. It also showed that a history of imprisonment, duration of injection, type of drug last injected and sharing needle/syringe were all significantly associated with HCV infection in all age groups. Sensitivity and specificity ranged from 89% and 16%, respectively, for cut-off ≥10 to 41% and 70%, respectively, for cut-off ≥25. Imprisonment and/or sharing needle/syringe were interestingly common items in the original EGCRISC as well as the EGCRISC-IDUs and EGCRISC-HIV.

A study in Baltimore, USA [19] was conducted among injecting drug users to develop and validate a brief screening tool for assessing the risk of contracting HIV among PWIDs. They developed an easy to administer seven-question screening tool with a cut-off that is predictive of incident HIV infection among injecting drug users. The AUC was 0.720; possible scores on index ranged from 0 to 100 and a score ≥46 had a sensitivity and specificity of 86.2% and 42.5%, respectively.

In this study, we developed a scoring tool for HCV in HIV patients (EGCRISC-HIV). History of imprisonment, male gender, low education, accidental puncture with protruding needle and alcohol use were associated with HCV infection among this group and were included in the developed tool. The tool had good operating characteristics that may help in the assessment of HCV risk in HIV patients.

### Limitations

This study has potential limitations. First, other potentially high-risk groups as prisoners, MSM and refugees due to inability to get the required approvals from the relevant authorities were not included. Applicability of the EGCRISC-HRG should be tested on these groups in future studies.

Second, the small number of females >45 years old in this study was found to be inconclusive. Further study is needed to test the EGCRISC-HRG on this group with a reliable sample size.

## Conclusion

This study introduced three risk assessment tools (EGCRISC-HRGs, EGCRISC-IDUs and EGCRISC-HIV). EGCRISC-HRG which comprises all high-risk groups is a valid tool that can be applied to high-risk populations. The specific EGCRISC-IDUs and EGCRISC-HIV are useful tools for preselecting potentially HCV-infected cases in the respective IDUs and HIV patients for further testing in settings where serological analyses are not readily available or accessible.

## References

[1] Anon. World Health Organization (WHO) (2016) Global Health Sector Strategy on Viral Hepatitis 2016–2021. Towards Ending Viral Hepatitis. Geneva, Switzerland: WHO.

[2] Petruzziello A (2016) Global epidemiology of hepatitis C virus infection: an up-date of the distribution and circulation of hepatitis C virus genotypes. World Journal of Gastroenterology 22, 7824–7840. 27678366 10.3748/wjg.v22.i34.7824 PMC5016383

[3] El-Zanaty F and Way A (2009) Egypt Demographic and Health Survey. Cairo, Egypt: Ministry of Health, El-Zanaty and Associates, and Macro International, In, 2008.

[4] El-Zanaty F and Way A (2015) Egypt Health Issue Survey. Cairo, Egypt: Ministry of Health and Population.

[5] MOHP (2014) Plan of Action for the Prevention, Care and Treatment of Viral Hepatitis, Egypt, 2014–2018. [online] Available at: http://www.emro.who.int/images/stories/Hepatitis_action_plan.pdf?ua=1 [Accessed 9 Sep. 2018].

[6] Doss W (2008) Egyptian National Control Strategy for Viral Hepatitis 2008–2012. Arab Republic of Egypt: Ministry of Health and Population, National Committee for the Control of Viral Hepatitis. Ministry of Health and Population, National Committee for the Control of Viral Hepatitis

[7] Elgharably A (2017) Hepatitis C in Egypt – past, present, and future. International Journal of General Medicine 10, 1–6. 28053553 10.2147/IJGM.S119301 PMC5191841

[8] El-Ghitany EM (2016) Validation of EGCRISC for chronic hepatitis C infection screening and risk assessment in the Egyptian population. PLoS ONE 11, e0168649. 28002458 10.1371/journal.pone.0168649 PMC5176306

[9] El-Ghitany EM (2019) Cost-effectiveness of EGCRISC application versus hepatitis C virus mass screening in Egypt. Journal of Infection and Public Health 12, 442–444. 30220579 10.1016/j.jiph.2018.08.004

[10] El-Ghitany EM, Farghaly AG and Farag S (2017) Performance of the validated EGCRISC screening tool in chronic hepatitis C infection detection after application in the Egyptian setting. Journal of Hepatology 66, S279–S280.

[11] El-Ghitany EM (2016) Toward a simple risk assessment screening tool for HCV infection in Egypt. Journal of Medical Virology 88, 1767–1775. 26970264 10.1002/jmv.24520

[12] Tariq H (2019) Hepatitis in slaughterhouse workers. World Journal of Hepatology 11, 37–49. 30705717 10.4254/wjh.v11.i1.37 PMC6354121

[13] Field A (2013) Discovering Statistics Using IBM SPSS Statistics. Sage.

[14] Leeflang MM (2013) Variation of a test's sensitivity and specificity with disease prevalence. CMAJ: Canadian Medical Association Journal 185, E537–E544. 23798453 10.1503/cmaj.121286 PMC3735771

[15] McGinn T (2008) Validation of a hepatitis C screening tool in primary care. Archives of Internal Medicine 168, 2009–2013. 18852403 10.1001/archinte.168.18.2009

[16] Zuure F (2010) Evaluation of a risk assessment questionnaire to assist hepatitis C screening in the general population. Euro Surveillance 15, 19539. 20429995

[17] Nguyen MT (2005) Description of a new hepatitis C risk assessment tool. Archives of Internal Medicine 165, 2013–2018. 16186472 10.1001/archinte.165.17.2013

[18] Wand H (2012) Developing and validating a scoring tool for identifying people who inject drugs at increased risk of hepatitis C virus infection. BMJ Open 2, e000387. 10.1136/bmjopen-2011-000387 PMC3253425 22218720

[19] Smith DK (2015) A brief screening tool to assess the risk of contracting HIV Infection among active injection drug users. Journal of Addiction Medicine 9, 226–232. 25961495 10.1097/ADM.0000000000000123 PMC4449303

